# Smartloss: A Personalized Mobile Health Intervention for Weight Management and Health Promotion

**DOI:** 10.2196/mhealth.5027

**Published:** 2016-03-16

**Authors:** Corby K Martin, L. Anne Gilmore, John W Apolzan, Candice A Myers, Diana M Thomas, Leanne M Redman

**Affiliations:** ^1^ Pennington Biomedical Research Center Baton Rouge, LA United States; ^2^ Montclair State University Montclair, NJ United States

**Keywords:** weight loss, app, eHealth, mHealth, SmartLoss, telehealth, mobile website, mobile phone

## Abstract

**Background:**

Synonymous with increased use of mobile phones has been the development of mobile health (mHealth) technology for improving health, including weight management. Behavior change theory (eg, the theory of planned behavior) can be effectively encapsulated into mobile phone-based health improvement programs, which is fostered by the ability of mobile phones and related devices to collect and transmit objective data in near real time and for health care or research professionals and clients to communicate easily.

**Objective:**

To describe SmartLoss, a semiautomated mHealth platform for weight loss.

**Methods:**

We developed and validated a dynamic energy balance model that determines the amount of weight an individual will lose over time if they are adherent to an energy intake prescription. This model was incorporated into computer code that enables adherence to a prescribed caloric prescription determined from the change in body weight of the individual. Data from the individual are then used to guide personalized recommendations regarding weight loss and behavior change via a semiautomated mHealth platform called SmartLoss, which consists of 2 elements: (1) a clinician dashboard and (2) a mobile phone app. SmartLoss includes and interfaces with a network-connected bathroom scale and a Bluetooth-connected accelerometer, which enables automated collection of client information (eg, body weight change and physical activity patterns), as well as the systematic delivery of preplanned health materials and automated feedback that is based on client data and is designed to foster prolonged adherence with body weight, diet, and exercise goals. The clinician dashboard allows for efficient remote monitoring of all clients simultaneously, which may further increase adherence, personalization of treatment, treatment fidelity, and efficacy.

**Results:**

Evidence of the efficacy of the SmartLoss approach has been reported previously. The present report provides a thorough description of the SmartLoss Virtual Weight Management Suite, a professionally programmed platform that facilitates treatment fidelity and the ability to customize interventions and disseminate them widely.

**Conclusions:**

SmartLoss functions as a virtual weight management clinic that relies upon empirical weight loss research and behavioral theory to promote behavior change and weight loss.

## Introduction

In the United States, over two-thirds of the adult population is classified as overweight or obese [1] and over 140 million US adults are eligible for weight loss treatment [2] based on the treatment guidelines published in 2013 [3]. Gold standard weight management programs are intensive, employ at least 14 in-person contacts in a 6-month period, and include: (1) a specific dietary goal or prescription, (2) self-monitoring of health indicators such as weight change, (3) individualized counseling that facilitates programmatic success in both the short-term (weight loss) and long-term (weight-loss maintenance), and (4) individualized feedback based on body weight change and dietary intake [3].

Intensive programs for weight management when delivered in-person have several limitations, including financial and geographical barriers, and can be cost prohibitive for many people. A less obvious but no less important barrier is that in-person interventions typically do not provide timely treatment advice to clients, which affects weight control. For example, a cornerstone of effective weight management is self-monitoring behaviors that track diet, physical activity, and weight. Behavior change and learning theory indicate that feedback by counselors that is both temporally contiguous and closely aligned with the client data being monitored produces superior behavior change and improved weight control [4]. Above all, in-person intensive programs for weight management deemed successful in large clinical trials have limited scalability. Dissemination of intensive in-person interventions to large numbers of individuals is costly and would significantly strain the health care system. Innovative and cost-effective treatment approaches for weight management that are grounded in behavior change theories need to be developed and tested.

With the insurgence of electronic and Internet-enabled devices available to consumers, the development of “online” or Internet-based programs for health improvement are more readily available. For weight management, mobile health (mHealth) interventions are particularly advantageous because, if designed appropriately, mHealth weight management programs overcome barriers for participation in traditional clinic-based treatments and thereby can reach many more consumers needing treatment. Indeed, Internet-based weight management interventions that are intensive (ie, include counselor support and individualized recommendations) produce clinically significant weight losses [5-8] that are comparable to intensive in-person interventions [9]. More scalable and automated programs, however, have produced much less weight loss.

Weight management programs delivered via remote devices (ie, mobile phones and tablets) are new to the mHealth technology domain. Mobile phones provide a platform for the collection of objective data from onboard or peripheral sensors and delivery of automated feedback, as well as counselor-driven feedback. The ability to rely on embedded communication platforms (eg, texting, phone calls) fosters synchronous rather than asynchronous feedback and communication. Mobile phones and tablets are nearly ubiquitous and allow people to be mobile yet stay connected to the Internet [10]. The number of devices doubles every 5 years [11]; in 2015, there were 3.5 devices for every person on the planet [12] and, by 2020, it is estimated that there will be approximately 50 billion devices worldwide [11]. Importantly, mobile phone-based programs present a promising approach to effectively reach individuals with limited access to health care. For example, underrepresented minorities are the most frequent users of mobile Internet [10] and households with low incomes are more likely to rely solely on mobile devices for Internet access rather than a computer [13].

In summary, given the increasing prevalence of overweight and obesity and the need for large numbers of individuals to be enrolled in effective treatment programs, cost-effective alternatives to intensive in-person programs need to be developed and tested. The aim of our work is to develop a virtual weight management program that can be deployed via mobile phones that: (1) provides individualized treatment goals; (2) allows for the collection of objective data from the client, as well as the input of self-reported data; (3) provides personalized feedback and treatment recommendations in near-real time; (4) delivers health information in a systematic fashion to foster healthy behavior changes; and (5) allows for remote monitoring of individuals by health care providers.

## Methods

### The SmartLoss Virtual Weight Management Suite

The SmartLoss Virtual Weight Management Suite includes 2 system components: a mobile application for mobile phones or tablets (the SmartLoss app) and a clinician dashboard. The SmartLoss app allows the end user to quickly receive information about their adherence to a prescribed diet and/or exercise goal, receive health information, enter data if needed, and foster synchronous communication with a health care professional.

The SmartLoss clinician dashboard is Internet-based and securely accessible by 3 levels of users: (1) administrators, (2) health care or research professionals, and (3) clients. Individual login IDs and passwords are required to access the SmartLoss clinician dashboard. Administrators have rights to: (1) create weight management programs for a clinic or group of clients; (2) assign a weight management program to a health care or research professional; (3) assign clients to a health care or research professional; (4) enter health materials for delivery during the weight management program; (5) enter delivery schedules for health information and the frequency of automated feedback; (6) view client data; and (7) generate reports that summarize client data at the level of the whole clinic/program, health care or research professional, or the individual client. Health care or research professionals have rights to: (1) view and enter weight and exercise (step) data for clients assigned to them, (2) generate client reports across all clients and also for a given individual, and (3) enter health materials and personalized feedback for delivery to clients. Clients have rights to view and enter only their own weight and step data, and to view health information specific to the weight management program or clinic. The SmartLoss clinician dashboard promotes treatment fidelity by providing real time access to intervention data.

### The Theoretical Framework of SmartLoss

Despite the ubiquity of mobile phones and peripheral devices to record health data, there is limited evidence that these devices and the information that they provide results in behavior change [14]. There are hundreds of weight management apps and, despite excitement over their promise [15,16], only a minority (15%) of apps incorporate evidence-based weight control methods [17]. Also, there is limited evidence for the efficacy of mHealth weight loss interventions [18-20] and the Guidelines for the Management of Overweight and Obesity in Adults [3] conclude that there is a low quality rating in the strength of evidence for mHealth interventions. Nonetheless, efficacy has been found for mHealth interventions such as SmartLoss and others that incorporate behavior change theory, self-monitoring, tracking of objective body weight data, and skills training [21-23].

SmartLoss is an ecological momentary intervention (EMI) or a program that delivers treatment to clients in their natural environment. EMIs rely on communication technology to deliver treatment, which clients find acceptable and effective at tracking progress toward objective goals [24]. SmartLoss utilizes remote devices to collect body weight and exercise information objectively and in near real time to facilitate timely behavior change. Specifically, learning theory [4] postulates that temporally contiguous feedback based on objective individual level data results in superior behavior change and fosters engagement of a client in treatment. Additionally, SmartLoss relies on the theory of planned behavior and the theory of reasoned actions [25] by fostering an environment conducive to behavior change where clients’ behavioral goals are clearly defined, self-efficacy is promoted, and behavior can be regulated through the presence of objective behavioral data. Clinic-based weight management interventions also rely on these theoretical frameworks, in addition to social cognitive theory [26]. SmartLoss incorporates aspects of social cognitive theory by reinforcing behavior change and fostering personal agency, clear outcome expectations, and goal setting, yet opportunities for modeling behaviors and vicarious learning are limited.

Development of weight loss and exercise goals is a collaborative process between the health care or research professional and the client, which facilitates client ownership of the behavioral goals. Further, the health care or research professional helps the client to develop positive attitudes and the intention to change behavior by providing specific behavioral changes that, if achieved, will result in a desired and expected outcome. Perceived behavioral control and self-efficacy are built as the client exerts self-control, makes behavioral changes, and experiences the positive consequences of behavior change (eg, weight loss, higher energy levels, compliments from others about weight loss, etc). Additionally, praise from the health care or research professional and automated feedback indicating that the client is on track to achieve established goals fosters behavior change, though intrinsic motivation increases over time as goals are internalized and self-efficacy and perceived behavioral control of the client is bolstered. It is recognized that motivation and perceived behavioral control naturally fluctuate during the course of treatment and health care or research professionals are able to utilize motivational interviewing [27] techniques, though such techniques are deployed remotely. As detailed below, the delivery of personalized treatment recommendations are based on objective data and are guided by an algorithmic approach that relies on a toolbox strategy, similar to those used in highly effective clinical trials of weight management [28-30].

### The SmartLoss Lifestyle Intervention

#### Tracking Body Weight as a Measure of Dietary Adherence

The SmartLoss lifestyle intervention approach is grounded in the ability to (1) determine the weight maintenance energy requirement for an individual, (2) establish realistic weight loss and dietary intake goals, (3) objectively quantify the adherence of an individual user to this weight loss and dietary prescription goal, and (4) from the individual data being inputted (ie, body weight) provide immediate and personalized feedback and treatment recommendations. Using data from adults in the National Health and Nutrition Examination Survey and adults who have completed highly controlled clinical trials that led to weight loss [31,32] or weight gain [33,34], we developed and validated dynamic differential equations based on the energy balance model [35-37]. We have demonstrated that these equations allow energy requirements of individuals to be accurately predicted from basic demographic and anthropometric data and, most importantly, the equations generate a prediction for the change in weight over the course of weight loss interventions that include an energy restricted diet and moderate levels of exercise. The model-predicted weight changes are then displayed graphically, which provides a guide for weight change throughout a given program. We further extended these mathematical models to provide accurate estimates of energy intake during weight change by inputting body weights over time [38]. Finally, we developed and validated a model that predicts weight change secondary to high levels of exercise only, and we developed and validated separate models that predict weight change during programs that include both an energy restricted diet and high levels of exercise [39]. The models form the cornerstone of the SmartLoss virtual weight management suite since, based on observed body weights, they provide the ability to quantify adherence to energy balance prescriptions induced by changing dietary intake and/or exercise behaviors.

**Figure 1:**
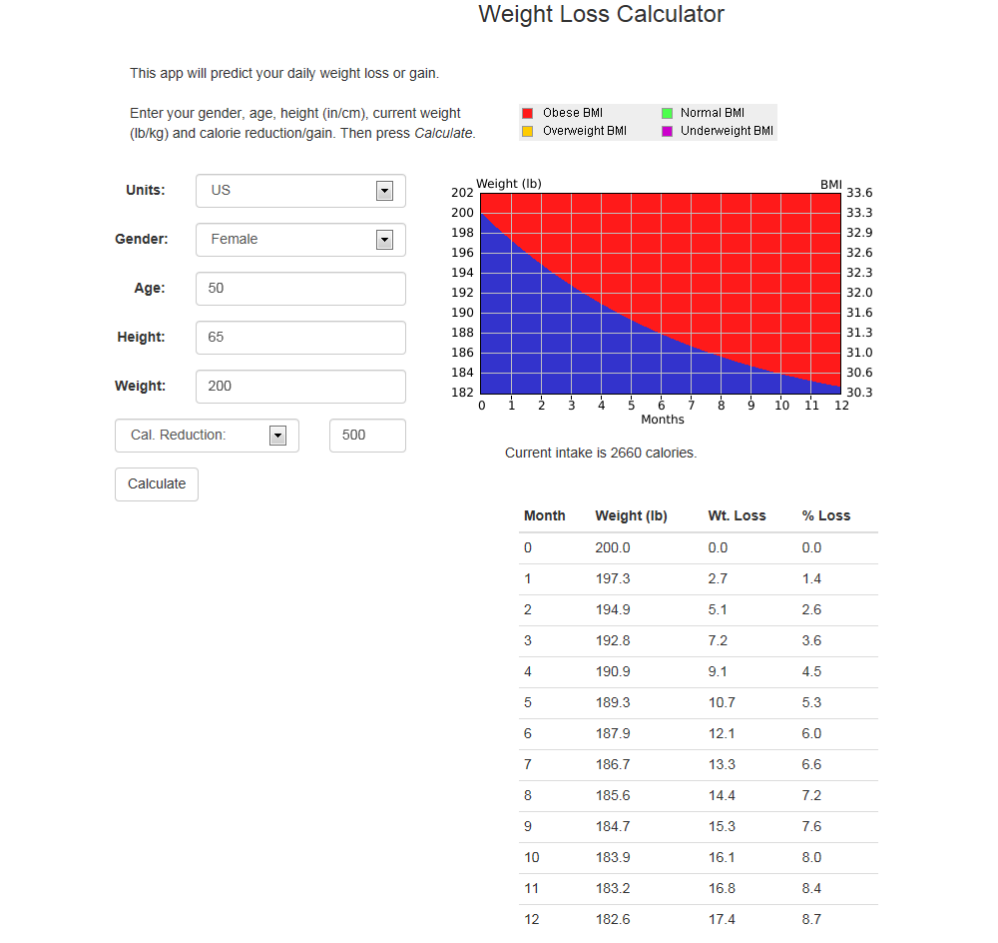
Predicted weight change based on mathematical models of energy balance and an energy intake target that reduces intake by 500 kcal/day. This example was created at http://weight-loss-predictor.appspot.com/weight for a hypothetical 50-year-old female who is 65 inches tall, weighs 200 pounds, and wishes to reduce her intake by 500 kcal/day, which would result in weight loss of 17.4 pounds or 8.7% over 12 months if adherent to the new energy intake level.

**Figure 2:**
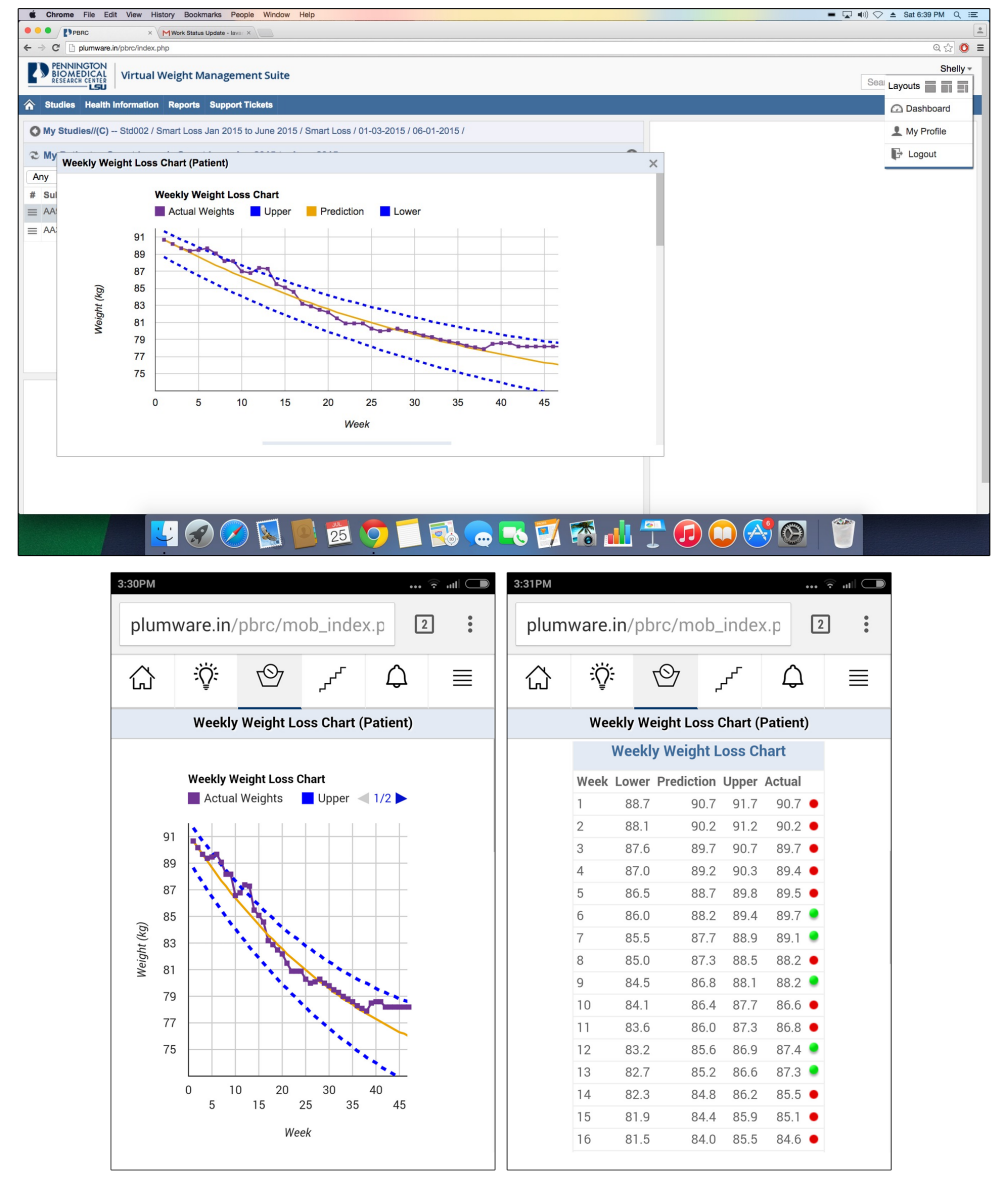
Panel A: A screenshot from the clinician dashboard for a hypothetical client. The SmartLoss intervention generates a SmartGraph that shows the predicted course of weight change for an individual assuming adherence to the energy intake target (yellow line), which can be either an increase or decrease in daily energy intake. The upper and lower bounds around the predicted weights (dashed blue lines) create a “weight zone” or “zone of adherence” and participants are considered adherent if their body weight (purple line) falls within the zone over time. Also, intervention strategies are delivered based on the participant’s actual weight in relation to this zone, and the graph is available to participants and health care or research professionals at any time via the clinician dashboard and app. Panel B: The SmartGraph is automatically delivered to participants smartphones at regular intervals (e.g., once per day, after a new body weight is received by the dashboard). Panel C: A “details view” in the app allows participants to see their weights over time in addition to their predicted weights, the weights associated with the upper and lower bounds of the adherence zone, and a color coded flag denoting if they were in or out of the zone (these same data are also available via the clinician dashboard).

The functionality of the mathematical models is demonstrated on the Weight Loss Calculator website (Figure 1) [40]. As shown in Figure 1, to use the models the user is required to enter sex, age, weight, and height. The user then can elect to either reduce or increase their calorie intake to view the effect of the energy balance prescription on their body weight. The example in Figure 1 is for a 50-year-old female who is 65 inches tall, weighs 200 pounds, and wishes to reduce her intake by 500 kcal/day. This example is for a client wishing to diet and not dramatically increase levels of exercise, yet exercise-only interventions and interventions that include both energy restriction and high levels of exercise can be accommodated, as detailed in the following section. In such cases, the weight graphs provide a proxy measure of adherence to the energy balance prescription, which promotes an energy imbalance of a specified size through changes to diet and exercise behaviors. As demonstrated in the output, this patient’s weight will decrease by 17.4 pounds or 8.7% over 12 months if adherent to her new energy intake level. This website is free to use, and the SmartLoss intervention relies on these mathematical models to generate a SmartGraph, which shows the predicted course of weight change for the individual as well as the acceptable upper and lower limits of the projected weight change for the chosen energy intake target. As demonstrated in Figure 2, the upper and lower bounds of the weight loss projection present a proxy for adherence to the energy balance prescription, which, in our example, is induced by reducing energy intake. The individual is considered adherent if, throughout the course of the weight management program, the change in body weight falls between the upper and lower bounds, which is termed the “weight zone” or “zone of adherence.” Intervention strategies, including personalized feedback, are delivered based on the actual weight of the individual in relation to this zone [21]. One of the strengths of this approach is the ability to objectively quantify adherence to diet and exercise recommendations based on observed body weight. As described herein, the SmartGraph is automatically created by SmartLoss and updated with new weight data in near real time, and it is viewable by the client and health care or research professional on the SmartLoss clinician dashboard and the app.

SmartLoss relies on weight data that can be automatically imported from commercially available devices. For example, SmartLoss is presently programmed with the Body Trace bathroom scale application program interface (API) to directly import body weight data. The bathroom scale synchronizes with the Internet via a cellular card, and body weight information is automatically and wirelessly transmitted to a server. The SmartLoss system retrieves weight data from the server in near real time and weight is plotted on the SmartGraph (Figure 2), which is available immediately via the SmartLoss app or the SmartLoss clinician dashboard. Additionally, weight data can be hand entered by clients via the mobile phone application or dashboard, or a health care or research professional can enter weight data into the SmartLoss clinician dashboard. Should a client weigh multiple times during the day or enter multiple daily weights, SmartLoss is programmed to plot only the first recorded weight of the day. It is also programmed to eliminate weights not obtained from the client (ie, weights that deviate by +/− 5% from the last available weight). The system is adaptable and the programming logic can be manipulated should a health care or research professional wish to evaluate weight fluctuations throughout the day, for example.

**Figure 3:**
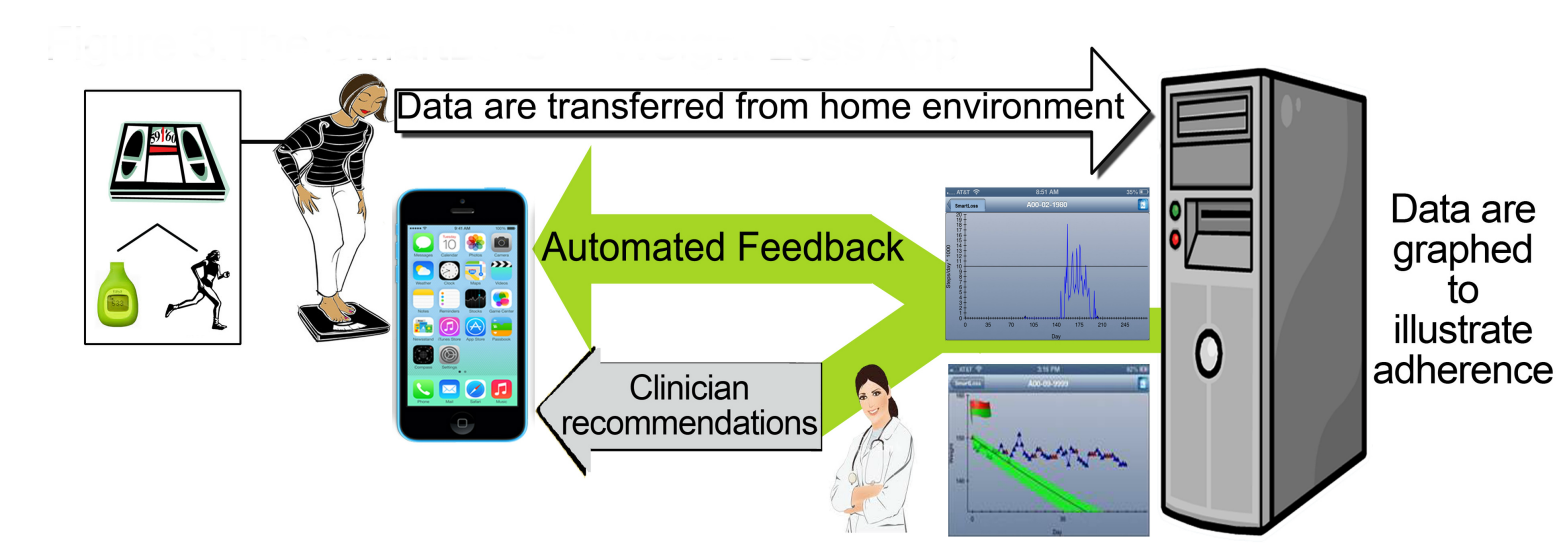
The overall concept of the SmartLoss Virtual Weight Management Suite: objective weight and exercise data are obtained, those data are integrated into personalized behavioral goals, and both automated and customized feedback based on client data and their relation to preestablished goals are transmitted to the client.

SmartLoss can transmit automated feedback to clients at prespecified intervals or based on weight change characteristics. For example, if the weight of a client is changing as expected (ie, is within the zone of adherence), the feedback includes a congratulatory message and a tip to encourage continued success. Via the clinician dashboard, the health care or research professional can have further interaction with clients by initiating additional personalized feedback messages and treatment advice. The collection of objective weight data (and exercise data, as described below) by clients, the integration of those data into personalized behavioral goals, and the delivery of both automated feedback and customized feedback generated by health care or research professional are illustrated in Figure 3. SmartLoss also provides instantaneous feedback to clients regarding the change in weight relative to the zone of adherence. The program automatically generates color-coded flags indicating if the weight of the client is: (1) within the zone of adherence (green flag); (2) within the zone but plateauing or approaching the upper edge of the zone (green/yellow flag); (3) out of the zone (red flag); or (4) above of the zone but decreasing at a rate that reflects adherence to the energy intake target (red/green flag) and, consequently, if continued will result in the client being back in the zone on the weight graph. If weight exceeds the target and is out of the zone for a given period of time (eg, 3 of 5 days), the client is provided with supportive treatment recommendations to modify energy intake and/or physical activity and foster adherence based on a toolbox approach, which is described below.

#### Tracking Client Adherence to Activity or Exercise Goals

**Figure 4:**
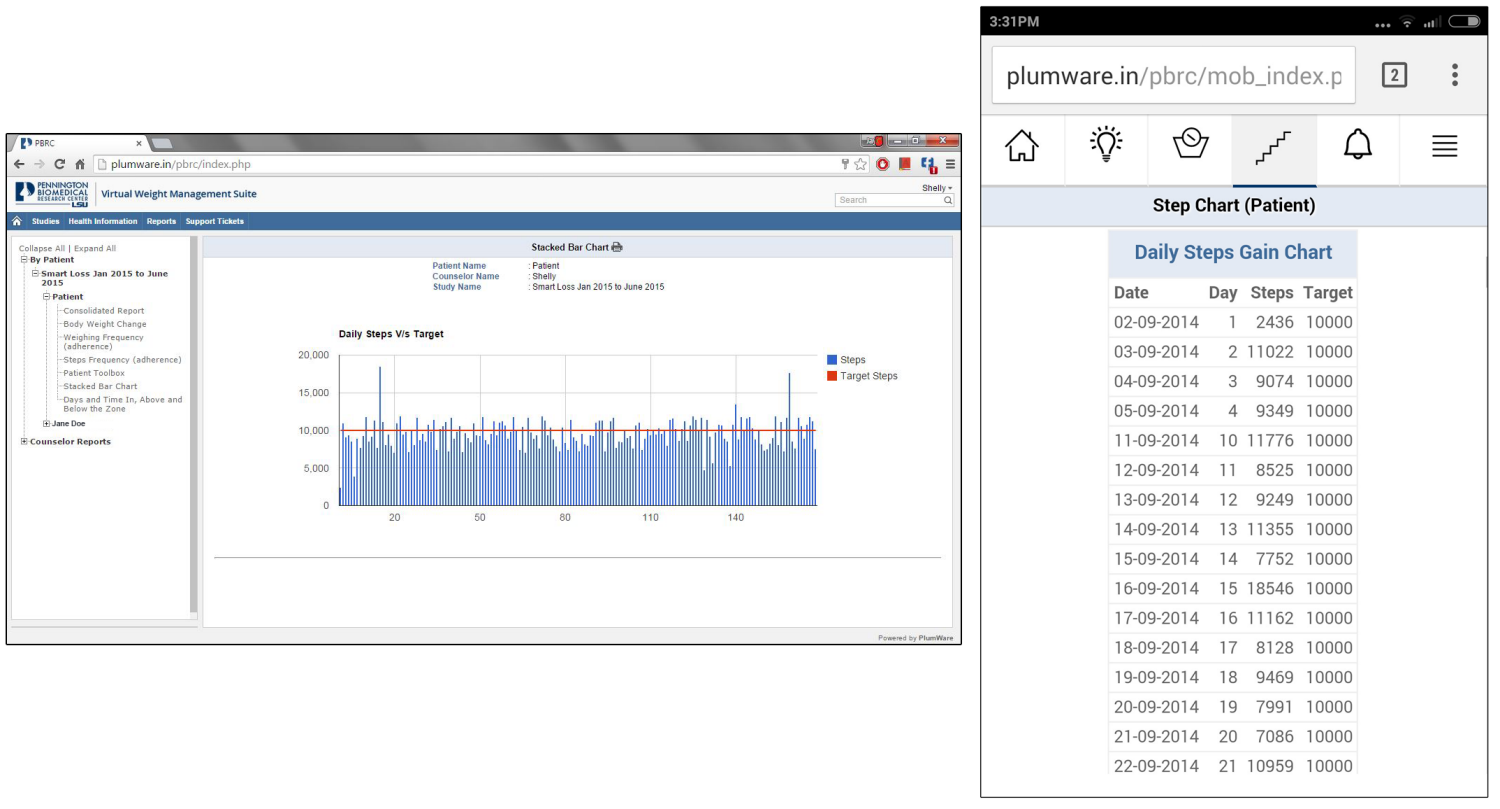
Panel A: A screenshot of the SmartSteps graph from the clinician dashboard, which is also delivered to clients’ mobile phones once per day. Panel B. The details view of a client’s step data on the smartphone app.

SmartLoss is programmed to automatically receive activity information from the FitBit API twice per day. Additionally, step data can be hand entered by participants via the SmartLoss app or dashboard, or a health care or research professional can manually enter weight data into the dashboard. After receiving activity data, the SmartLoss program summarizes the activity goal of the client and current steps per day on a SmartSteps graph for immediate viewing within the mobile phone app and clinician dashboard (Figure 4).

Adoption of a regular program of physical activity is important for weight management and exercise was considered when developing the energy balance models that predict weight loss; hence, the models include a term for activity [35-37]. The model currently used by SmartLoss produces weight loss predictions that are valid for individuals engaging in light to moderate levels of physical activity at the federally recommended level of 150 minutes per week [41]. Should an intervention rely only on higher levels of exercise, a different model that relies on a modified Forbes curve would be used to predict weight change [39]. Similarly, if an intervention promoted a combination of higher levels of exercise and an energy-restricted diet, an alternative model would be used to predict weight change that includes inputs for exercise and restriction of energy intake.

The physical activity component of SmartLoss is designed to encourage individuals to adopt a regular program of physical activity with the goal of increasing their daily steps by 3000 to 4000 steps per day above baseline [42], which results in 7000 to 8000 steps per day [41,43] and is consistent with federal guidelines of 150 minutes per week of moderate intensity activity. Since very few activity monitors provide valid estimates of caloric expenditure, the focus of the exercise goals in SmartLoss is steps per day. Similar to feedback of weight data, individuals receive automated feedback messages about daily adherence to the physical activity goals. Long-term weight loss maintenance is promoted by higher levels of exercise [41,44], and elevated levels of exercise can be promoted and monitored over the long-term through SmartLoss, particularly after weight is lost and clients enter a weight maintenance phase.

#### SmartTips

SmartLoss can deliver a comprehensive set of health materials called SmartTips to educate clients on strategies for effective behavior change and weight management. A key feature of intensive in-person sessions is the receipt of health information to motivate and inform lifestyle change. During in-person interventions, these materials are generally printed and a health care or research professional reviews this information with clients in group or individual sessions and works with clients to apply the material to their own situation in an attempt to facilitate and maintain behavior change. The content of the SmartTips can be customized for different groups of individuals (eg, post-menopausal women, young men, lactating women) or with a focus toward a particular type of diet (ie, DASH diet, Mediterranean diet, etc). The interval at which the SmartTips are delivered can be customized for individual clients or programs, but participation in intensive intervention programs generally warrants receipt of health information weekly at the onset of the program and can taper off to biweekly and then monthly as the program progresses.

The SmartTips also provide a platform to reinforce and facilitate the use of strategies that effectively promote weight management. For example, use of portioned-controlled foods is an effective strategy in weight management programs and links can be embedded in the SmartTips to direct clients to credible websites where information on a variety of portioned-controlled foods can be obtained. The SmartTips are also interactive, requiring input from the client that acknowledges comprehension of the content. SmartTips also reference the SmartGraphs and recent weight change of the client. SmartLoss is therefore adaptable to many different intervention approaches, from purely automated delivery of SmartTips to the delivery of SmartTips with a counselor remotely reviewing the material with the client via a mobile phone’s multimedia capabilities. Thus, similar to in-person intensive lifestyle interventions, SmartLoss provides a platform for health care or research professionals to customize intervention delivery to the needs of individual clients and facilitate behavior change on an individual level. Further, SmartLoss provides a platform for researcher professionals to test the effects of remotely delivered interventions that include counselor support versus interventions that are fully automated but otherwise identical. Such research is needed, as SmartLoss and similar interventions that have been found to be efficacious were also fairly intense [21-23], while less intense and less directive interventions have produced less weight loss [45,46].

#### Toolbox Options

When body weight is repeatedly outside the zone of adherence, it serves as an objective indicator to the client and health care or research professional that more intensive treatment strategies are needed. Different treatment strategies to increase the intensity of the weight management program are maintained in the SmartToolBox. Examples of these strategies include the use of portion controlled foods, increased frequency of contact with the assigned health care or research professional, increased activity or exercise, adoption of a plan to self-monitor food intake, etc. This toolbox approach is similar to the strategy used in the Comprehensive Assessment of Long-term Effects of Reducing Intake of Energy (eg, the CALERIE study) [30,47] and other studies (eg, LookAhead) [28], and it provides a systematic and algorithmic method to improve adherence to diet and weight goals.

**Figure 5:**
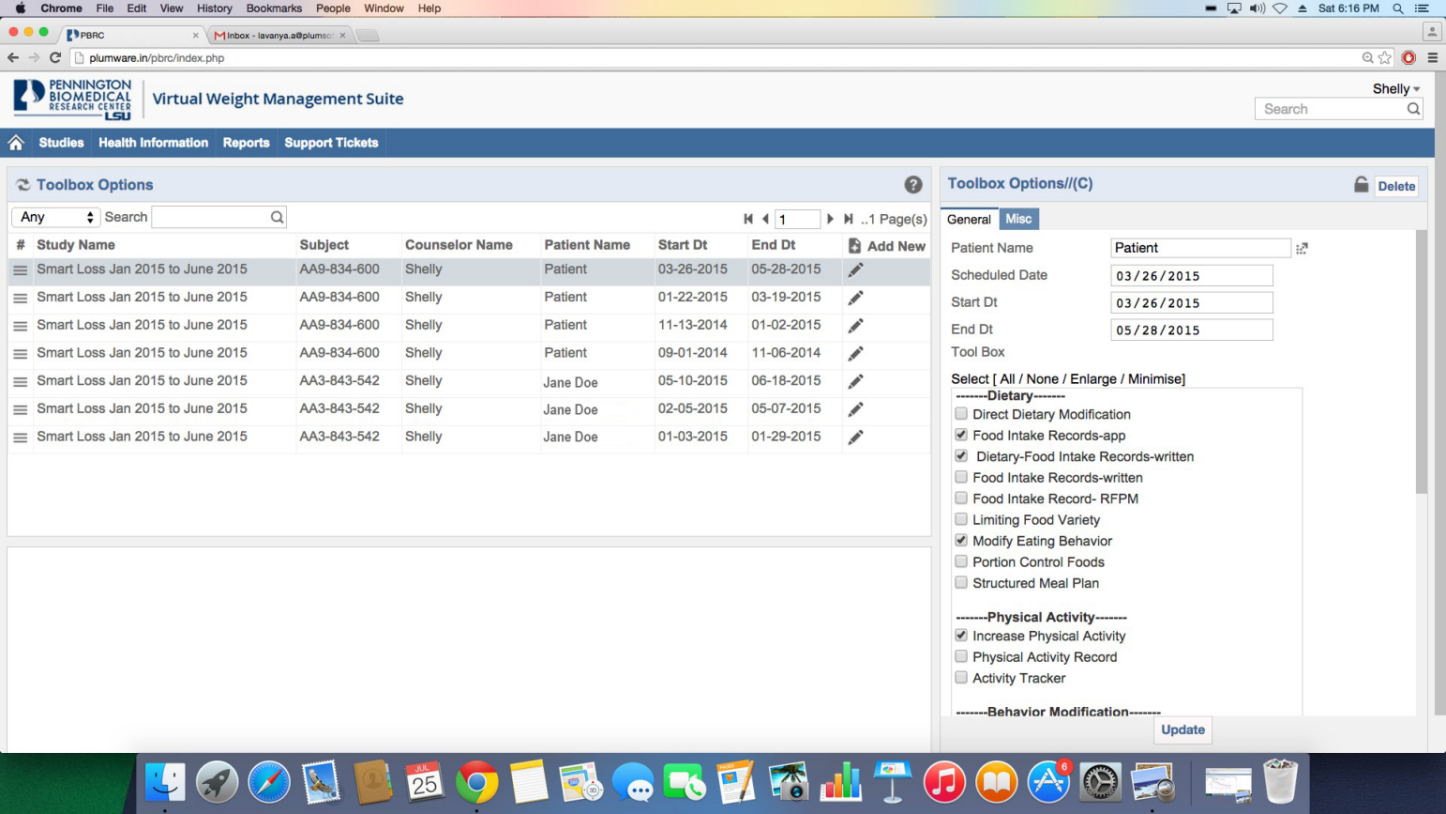
Screenshot of the SmartToolbox section of the clinician dashboard. SmartToolbox options are utilized when clients require additional support to meet their goals. Less intense and usually more affordable options are used first and progress is tracked to determine if improvement occurred. If not, more intense and usually more costly options are utilized and progress is similarly tracked. The clinician dashboard automatically tracks toolbox use and reports can be generated to demonstrate which options were utilized, as well as change in weight and exercise levels during use of the different toolbox options.

When using this approach, the client and health care or research professional are prompted by the SmartLoss app and dashboard to first select less intensive strategies from the toolbox, followed by more intensive and frequently more expensive strategies if the less intense strategies fail to result in the desired outcome (usually weight loss) over a given period of time (eg, 2 weeks). Treatment strategies provided within the SmartToolBox are specified by the clinic and customized for the type of weight management program being delivered and to meet the needs of individual clients (Figure 5). Use of the toolbox is tracked and quantified by the clinician dashboard. One aim of the toolbox approach is to build self-efficacy as selection of toolbox strategies is a collaborative process between the client and their healthcare or research professional, particularly early in treatment. Options are personalized to meet the individual client’s needs based on their current circumstances and abilities. As treatment progresses, the client learns to identify potential problems, evaluate and select a strategy to overcome the problem, and track the success of the strategy. This framework follows an active problem-solving approach and builds accountability and self-efficacy since the client is actively involved in choosing methods to improve adherence, evaluating the effectiveness of the chosen solution, and selecting an alternative solution if needed.

#### Usage Tracking and Report

The clinician dashboard allows users with administrator and healthcare or research professional rights to generate reports that summarize data on usage of the SmartLoss app, as well as reports showing weight and exercise data at the individual and group level. Usage data includes: (1) the number of days that body weight and step data were recorded, (2) the number of days clients were enrolled in the program, (3) the percent of days with weight and step data, (4) the number of times participants viewed the weight and step graphs, and (5) the number of times and which SmartTips were viewed. Reports can also be generated that summarize the following body weight data: (1) client body weight over time, (2) the target/goal weight and the upper and lower bounds of the zone of adherence, (3) client weight change (pounds and percent) over time, and (4) the number of days and percent of time that clients were in, below, or above the zone of adherence. Finally, reports can be generated that summarize SmartToolBox use and weight change and step counts during the time that different toolbox options were utilized. All reports can be viewed within the clinician dashboard and the raw data can be downloaded into other software packages for analysis.

## Results

Evidence of the efficacy of the SmartLoss approach has been reported previously [21]. The present report provides a thorough description of the SmartLoss Virtual Weight Management Suite, a professionally programmed platform that facilitates treatment fidelity and the ability to customize interventions and disseminate them widely.

## Discussion

Mobile phones and similar Internet-enabled devices (eg, tablets) are virtually ubiquitous and provide the opportunity to deliver weight management and health promotion programs to people with limited access to health care. Nonetheless, the preponderance of mobile phones and peripheral devices that provide objective health information to the user and, frequently, their clinician has failed to result in a large number of efficacious mHealth weight management interventions. SmartLoss is an EMI that quickly provides participants with feedback about their adherence to energy intake and activity goals via graphical displays. Importantly, adherence to the weight management program is quantified based on actual changes in client body weight and validated mathematical models of energy balance, and SmartLoss relies on the established behavioral theories to initiate and sustain behavior change.

SmartLoss and similar interventions [22,23] address shortcomings identified through earlier research, including the need to incorporate behavior change theory, self-monitoring, tracking of objective body weight data, and skills training. Based on the literature, it appears clear that more intense mHealth interventions such as SmartLoss are more efficacious at reducing body weight while more passive interventions are not as effective [45,46]. An unanswered question is the extent to which efficacy can be maintained during longer-term weight management interventions that increasingly rely on automated feedback versus feedback and treatment recommendations from a health care or research professional who delivers treatment remotely. Additionally, it seems likely that tracking objectively measured body weight during treatment is important, yet it is unclear to what extent interventions can maintain efficacy and rely on self-reported data. The ability of mHealth interventions to promote long-term weight loss maintenance also is not known and research on their efficacy and cost-effectiveness at promoting weight loss maintenance is warranted.

In conclusion, the SmartLoss Virtual Weight Management Suite provides health care providers and researchers with the ability to deliver weight loss and weight maintenance treatment plans to individuals remotely via a mobile phone app and an Internet-based clinician dashboard. It is hoped that this and similar theoretically-based mHealth interventions will facilitate the delivery of effective health promotion programs to populations who are typically underserved or who face barriers to engaging in high intensity programs offered in urban clinical settings.

## Abbreviations

API: application program interface
EMI: ecological momentary intervention
mHealth: mobile health
